# Mutation of the novel acetylation site at K414R of BECN1 is involved in adipocyte differentiation and lipolysis

**DOI:** 10.1111/jcmm.16692

**Published:** 2021-06-04

**Authors:** Chengqian Li, Jun Xu, Qing Yu, Ping Wang, Bingzi Dong, Liyan Shen, Qing Wang, Shufa Li, Ying Yang, Yujie Deng

**Affiliations:** ^1^ Department of Endocrinology and Metabolism The Affiliated Hospital of Qingdao University Qingdao China; ^2^ Department of Geriatrics Shanghai JiaoTong University Affiliated Sixth People's Hospital Shanghai China; ^3^ Shanghai Key Laboratory of Diabetes Shanghai Institute for Diabetes Shanghai Clinical Medical Centre of Diabetes Shanghai Key Clinical Centre of Metabolic Diseases Department of Endocrinology and Metabolism Shanghai JiaoTong University Affiliated Sixth People's Hospital Shanghai China

**Keywords:** acetylation, adipocytes, autophagy, BECN1, differentiation, lipolysis

## Abstract

BECN1, a protein essential for autophagy, is involved in adipocyte differentiation, lipolysis and insulin resistance. The discovery of new mechanisms for modifying BECN1 in adipocytes may provide novel therapeutic targets for obesity. This study aimed to investigate the impact of mutations at the acetylation sites of BECN1 on adipocyte differentiation and lipolysis. We found that Ace‐BECN1 levels were increased in 3T3‐L1 adipocyte differentiation and isoproterenol‐/TNF‐α‐stimulated lipolysis and in subcutaneous and visceral adipose tissues of high‐fat diet mice. K414 was identified as an acetylation site of BECN1, which affects the stability of the BECN1 protein. Mutation at K414 of BECN1 affected autophagy, differentiation and lipolysis in 3T3‐L1 adipocytes. These data indicated the potential of BECN1 K414 as a key molecule and a drug target for regulating autophagy and lipid metabolism in adipocytes.

## 1 INTRODUCTION

Macroautophagy (hereafter referred to as autophagy) is a phenomenon that exists in all eukaryotic cells. It involves the degradation and recycling of damaged or ageing organelles and macromolecules through the formation of a bilayer membrane structure, the autophagosome.^1^ Autophagy protects the cells and has to be perfectly regulated to respond adequately to the cell microenvironment, stimuli and threats.^1^ Dysregulated autophagy is involved in a number of diseases; however, most studies have focused on the involvement of autophagy in cancer and neurodegeneration,^1^ and its role in lipid metabolism gained interest only a few years. Singh et al suggested that lipid droplets (LD) can be degraded in hepatocytes through the autophagic pathway, known as macrolipophagy or lipophagy.^2^ Autophagy was also demonstrated to regulate adipogenesis. Knockdown of Atg7 in 3T3‐L1 pre‐adipocytes inhibited lipid accumulation, and adipocyte‐specific Atg7‐null mice showed decreased white adipose mass,^3^, ^4^ suggesting that autophagy is required for the development of white adipose tissue. Controversially, Soussi et al showed attenuated autophagic activity in human obesity.^5^ These authors suggested that, in addition to detecting mRNA or protein levels of ATG genes or autophagy regulators, autophagic clearance should also be measured according to published autophagy guidelines.^6^ Therefore, monitoring autophagic flux is important for the analysis of autophagy. The lysosomal inhibitor chloroquine (CQ) can be used to evaluate autophagic flux.

The regulation of autophagy is highly complex, and a large number of effector proteins are involved in the process, many of which belong to the Atgs (autophagy‐related genes).^7^ BECN1 is an essential Atg for the regulation of autophagosome formation and maturation by forming distinct PI3K complexes. BECN1 functions as a tumour‐suppressor gene, and its deficiency is also associated with several neuro‐degenerative diseases. Previous studies by our group suggested that BECN1 is required for autophagy in 3T3‐L1 mature adipocytes.^8^ The functions of Beclin1 involved in autophagosome biogenesis can be regulated by post‐translational modifications, which include phosphorylation, ubiquitination, acetylation, proteolytic cleavage and O‐Linked β‐N‐acetylglucosamine modification.^9^ The modifications affect its stability, conformation, activity and its interactome. Qian et al demonstrated that glutamine deprivation and hypoxia lead to phosphoglycerate kinase 1‐mediated Beclin1 S30 phosphorylation. This phosphorylation enhances ATG14L‐associated class III phosphatidylinositol 3‐kinase VPS34 activity by increasing the binding of phosphatidylinositol to VPS34, which are required for glutamine deprivation‐ and hypoxia‐induced autophagy and brain tumorigenesis.^10^ Acetylation is an important post‐translational modification of proteins. However, there is currently only one study on BECN1 acetylation in tumour cells^11^ and one on Alzheimer's disease,^12^ and no studies are available regarding BECN1 acetylation in adipocytes. BECN1 can be acetylated and deacetylated by p300 and sirtuin 1 (SIRT1) at K430 and K437, and acetylation of BECN1 inhibits autophagosome maturation.^11^ BECN1 acetylation in Alzheimer's disease neurodegeneration impairs the autophagic flux.^12^ To investigate BECN1 acetylation in mature adipocytes will offer new insight into the regulation of adipocyte autophagy and provide a basis for the study of the role of autophagy in lipid metabolism. It could also provide novel therapeutic targets for obesity and T2DM.

The present study found that Beclin1 could be acetylated in 3T3‐L1 mature adipocytes. Acetylation of BECN1 at the K414 site affected autophagy and autophagic flux, and may be involved in the differentiation (pre‐adipocytes to mature adipocytes) and lipolysis of adipocytes.

## 2 METHODS

### 2.1 Cell lines and animals

The 3T3‐L1 pre‐adipocytes and HEK293 cells were obtained from Aspen (China). Healthy male 4‐week‐old C57BL/6 mice weighing 15‐20 g were purchased from Hubei Experimental Animal Research Center (licence number: SCXK (E) 2015‐0018).

### 2.2 Adipocyte differentiation

The 3T3‐L1 pre‐adipocytes were seeded onto a 6‐well plate at 10^5^ cells/well. Complete culture medium was added, and the cells were cultured at 37℃ with 5% CO_2_. The medium was changed every 2 days. After 2 days, complete culture containing 0.5 mM 3‐Isobutyl‐1‐methylxanthine (IBMX, I5879; Sigma‐Aldrich, St. Louis, MO, USA ), 1 µM dexamethasone (D4902; Sigma‐Aldrich) and 5 µg/mL insulin (HI0240; Eli Lilly, Indianapolis, IN, USA) were added (2 mL/well), and the cells were incubated for 48 h. The medium was replaced with a complete culture medium containing 5 µg/mL insulin (2 mL/per well). After 48 h, culture was continued using the complete culture medium, and the medium was changed every 2 days. At 8‐10 days after induced differentiation, 80%‐90% of 3T3‐L1 pre‐adipocytes showed an adipocyte phenotype. Morphology was examined over 10 days using oil red O staining and light microscopy (Nikon, Tokyo, Japan).

### 2.3 BECN1 overexpression

The BECN‐1 gene was obtained, and the BECN1‐Flag‐pLVX‐ZsGreen1‐N1 vector was constructed. One day before transfection, 1 × l0^5^ HEK293 cells were seeded in a 24‐well plate. Transfection required a cell confluence of 90%–95%. The cells were grouped as follows: the empty transfection group (empty plasmid) and the transfection group (BECN1 overexpression plasmid). The DNA was diluted with 50 μL of OPTI‐MEM medium. Then, 2 μL of Lipofectamine 2000 (11668‐019, Invitrogen Inc, Carlsbad, CA, USA) was diluted with 48 μL of Opti‐MEM low serum medium and incubated at room temperature for 5 minutes. The DNA and Lipofectamine mixtures were mixed together and left at room temperature for 20 minutes. The cells were washed twice with OPTI‐MEM medium and resuspended in 400 μL of OPTI‐MEM medium. The DNA mixture was added and mixed thoroughly. The cells were incubated in a CO_2_ incubator at 37℃ for 48 hours to detect protein and gene expression. Transfection was confirmed by western blotting and RT‐PCR.

### 2.4 Mouse model establishment and grouping

Mice were randomly assigned to two groups: normal diet and high‐fat diet (HFD). The mice in each group were free to eat and drink water, and their weight was measured once a week. After 3 months of continuous feeding (weight of the obese mice had to be ≥50 g), the mice were killed by cervical dislocation. The subcutaneous fat and visceral fat (epididymal fat) tissues were isolated, snap‐frozen and kept at −80℃.

### 2.5 Western blot

Total proteins in the cells were extracted using RIPA buffer. Protein concentration was determined using a BCA protein concentration detection kit. The proteins were mixed with 5× protein loading buffer and denatured in a boiling water bath for 5 minutes. The proteins were separated using 10% or 12% polyacrylamide gel electrophoresis and transferred to PVDF membranes. The membranes were incubated overnight at 4℃ with the primary antibodies: rabbit BECN1 (#3495; 1:2,000, Cell Signaling Technology, Danvers, MA, USA), rabbit LC3 (#4108; 1:1,000, Cell Signaling Technology), rabbit CEBPα (ab40764; 1:1,000, Abcam, Cambridge, UK), rabbit aP2 (ab92501; 1:1,000, Abcam), rabbit PPARγ (ab209350; 1:500, Abcam), rabbit FAS (ab82419; 1:1,000, Abcam), rabbit HSL (ab45422; 1:500, Abcam), rabbit p‐HSL(ser563) (PA5‐17488; 1:1,000, Thermo Fisher Scientific, Waltham, MA, USA), rabbit p‐HSL(ser565) (PA5‐17487; 1:1,000, Thermo Fisher Scientific), rabbit p‐HSL(ser660) (bs‐3358R; 1:500, Bioss Antibodies, Woburn, MA, USA), rabbit ATGL (ab109251; 1:1,000, Abcam), rabbit perilipin (NB110‐40760; 1:1,000, Novus Biologicals, Littleton, CO, USA) and rabbit GAPDH (ab37168; 1:10,000, Abcam). The HRP‐conjugated goat anti‐rabbit secondary antibody (AS1107; 1:10,000, Aspen, China) was incubated at room temperature for 30 minutes. Electrochemiluminescence (ECL; Pierce Chemical, Dallas, TX, USA) was used to reveal the bands. The film was scanned and archived, and the AlphaEaseFC (Alpha Innotech, San Leandro, CA, USA) processing system was used to analyse the optical density value of the target band.

### 2.6 Immunoprecipitation (IP)

Total proteins in the cells were extracted using RIPA buffer. Protein concentration was determined using a BCA protein concentration detection kit. The BECN1 antibodies (#3495; 1:2,000, Cell Signaling Technology) were incubated in RIPA buffer, and magnetic beads were added. The unbound proteins were removed by centrifugation, and the bound proteins (immune complexes) were eluted. Western blotting was used to analyse the proteins, as above.

### 2.7 Real‐time polymerase chain reaction (RT‐PCR)

After washing the cells with pre‐cooled PBS, total RNA was extracted using TRIzol solution (Invitrogen, Carlsbad, CA, USA). The SYBR^®^ Premix Ex Taq™ kit (DRR081A, Takara, Japan) was used for detection on the StepOne™ Real‐Time PCR amplifier (Life Technologies, Carlsbad, CA, USA). All experiments were independently performed three times in triplicate. The primer sequences were as follows: *Becn1,* sense, 5′‐GGAATGAAATCAATGCTGCCT‐3′, antisense, 5′‐CCCCAGAACAGTATAACGGCA‐3′; GAPDH, sense, 5′‐TGAAGGGTGGAGCCAAAAG‐3′, antisense, 5′‐AGTCTTCTGGGTGGCAGTGAT‐3′.

### 2.8 BECN1 acetylation

The cells were treated with the deacetylase inhibitor nicotinamide (NAM) 5 mM and the histone deacetylase inhibitor TSA 10 µM for 12 hours.^11^ Ace‐BECN1, BECN1 and LC3 were tested using Western blots and immunoprecipitation.

### 2.9 Construction of *Becn1* mutation plasmid

The *Becn1*(K414R) gene was cloned and the primer sequences were as follows: sense, 5′‐ GGCCGCCACCAAGCTTGGTACCATGGAGGGGTCTAAGGCGTCCAGC‐3′; antisense, 5′‐ CATACCGGTCTTAAGGTTAACGGATCCCTTGTTATAGAACTGTGAGGACAC ‐3′, each of which contained HindIII (1060, Takara, Japan) or BamHI (1010, Takara, Japan) cutting sites, respectively. The *Becn1*(K414R)‐pLVX‐ZsGreen1‐N1 mutant vector was constructed and packaged in lentiviruses. Cells were grouped as follows: the wild‐type (WT) group (expressing WT BECN1) and mutated (MT) group (expressing K414R BECN1). One day before the experiment, 5 × 10^4^ cells/well in 100 μL were seeded in 96‐well culture plates and cultured to 40%‐60% confluence. Following incubation for 24 hours, an appropriate amount of viruses at a multiplicity of infection (MOI) value of 80 was added, and culture medium was added to 500 μL. Polybrene (0.5 μL, 1 mg/mL) was added to each well. The final polybrene concentration in the cell sample was 5 μg/mL. After mixing well, the cells were incubated in a 37℃, 5% CO_2_ incubator for 72 hours.

### 2.10 Transmission electron microscopy

The cells in each group were collected, centrifuged and fixed in 2.5% glutaraldehyde in 0.1 M sodium cacodylate buffer (pH 7.4), and washed three times with 0.1 M phosphate buffer. Osmic acid (1%) and phosphate‐buffered saline (PBS, 0.1 M, pH 7.4) were used for fixation at room temperature (20℃). The cells were embedded in epoxy resin, sectioned and examined by transmission electron microscopy (JEOL, Tokyo, Japan).

### 2.11 Immunofluorescence

The cells were grouped as follows: the wild‐type (WT) group, WT +chloroquine group (60 µM, 12 hours),^8^ MT group and MT +chloroquine group (60 µM, 12 hours), and were cultured in 6‐well plates. The culture supernatant was discarded. PBS was used to wash the cells three times, and 4% paraformaldehyde was used for fixation for 30 minutes at room temperature with a permeabilization working solution. The cells were treated with 3% H_2_O_2_ in the dark for 20 minutes. The rabbit LC3 (#4108; 1:200, Cell Signaling Technology) antibody was incubated at 4℃ overnight. The secondary antibody (AS‐1110, 1:50, Aspen, China) was incubated at 37℃ for 50 minutes. DAPI staining solution was incubated at room temperature for 5 minutes. The cells were observed under a confocal microscope (IX51, Olympus; Tokyo, Japan).

### 2.12 Lipolysis assay and ELISA

The cells were grouped as follows: WT group, WT + isoproterenol (ISO, 1 µM, 30 minutes)^13^ group, WT + TNF‐α group (50 ng/mL, 3 h),^13^ MT group, MT + ISO group and MT + TNF‐α group. Western blotting was performed for GAPDH, FAS, HSL, P‐HSL(ser563), P‐HSL(ser565), P‐HSL(ser660), ATGL and perilipin. Glycerin (F005‐1, Nanjing Jiancheng Biotech, Beijing, China), NEFA (A032, Nanjing Jiancheng Biotech), leptin (ELK1234; ELK Biotechnology, Wuhan, China) and adiponectin (ELK1161; ELK Biotechnology) in the cell supernatant were tested according to the manufacturers' instructions.

### 2.13 Statistical analysis

All data are shown as the mean ± standard deviations of three independent experiments performed in triplicate. Data were analysed using the two‐tailed Student's *t* test or one‐way ANOVA. Significance was shown as ^*^ *P* < .05, ^**^ *P* < .01 or ^***^ *P* < .001.

## 3 RESULTS

### 3.1 Ace‐BECN1 levels increased during adipocyte differentiation and lipolysis

**FIGURE 1:**
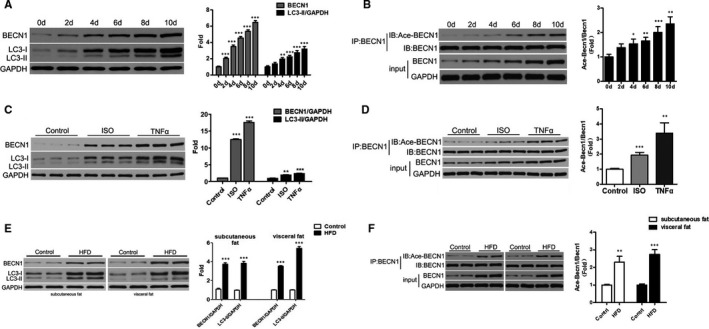
Ace‐BECN1 levels were increased during adipocyte differentiation and lipolysis. Pre‐adipocytes 3T3‐L1 were induced for up to 10 days. Western blot showed increased expression of BECN1 and LC3‐II proteins over time (A). Immunoprecipitation (IP) showed that Ace‐BECN1 levels were increased over time (B). When 3T3‐L1 mature adipocytes were treated with isoproterenol (ISO) 1 µM for 30 min and TNF‐α 50 ng/mL for 3 h, Western blot showed increased expression of the BECN1 and LC3‐II proteins over time (C). When 3T3‐L1 mature adipocytes were treated with ISO 1 µM for 30 min and TNF‐α 50 ng/mL for 3 h, IP suggested that Ace‐BECN1 levels were increased (D). Subcutaneous and visceral adipose tissues of normal and obese mice (≥50 g, n = 6 for each group) were obtained after 3 months of high‐fat diet (HFD) to detect the levels of BECN1 and LC3. BECN1 and LC3 were elevated in the subcutaneous and visceral fat of obese mice (E). IP results showed increased levels of Ace‐BECN1 in the subcutaneous and visceral adipose tissues of high‐fat diet (HFD) mice (n = 6) (F). Data are shown as the means ± standard deviations of three independent experiments. ^*^ *P* < .05, ^**^ *P* < .01 or ^***^ *P* < .001

**The** 3T3‐L1 pre‐adipocytes were induced for up to 10 days. At 8‐10 days after induced differentiation, 80%–90% of 3T3‐L1 pre‐adipocytes showed an adipocyte phenotype. Western blot showed increased expression of BECN1 and LC3‐II proteins over time (Figure 1A). IP showed that Ace‐BECN1 levels were increased over time (Figure 1B). When 3T3‐L1 mature adipocytes were treated with ISO 1 µM for 30 minutes and TNF‐α 50 ng/mL for 3 hours, Western blot showed increased expression of the BECN1 and LC3‐II proteins over time (Figure 1C), while IP suggested that Ace‐BECN1 levels were increased (Figure 1D). The subcutaneous and visceral adipose tissues of normal and HFD mice (≥50 g) were obtained to detect the levels of BECN1 and LC3, and it was found that BECN1 and LC3 were elevated in these tissues of HFD mice (*P* < .001) (Figure 1E). IP showed increased levels of Ace‐BECN1 in the subcutaneous and visceral adipose tissues of HFD mice (Figure 1F). Taken together, these results suggest that Ace‐BECN1 may play a role in adipocyte differentiation and lipolysis.

### 3.2 BECN1 is acetylated by NAM and TSA in both 293T cells and 3T3‐L1 adipocytes

**FIGURE 2:**
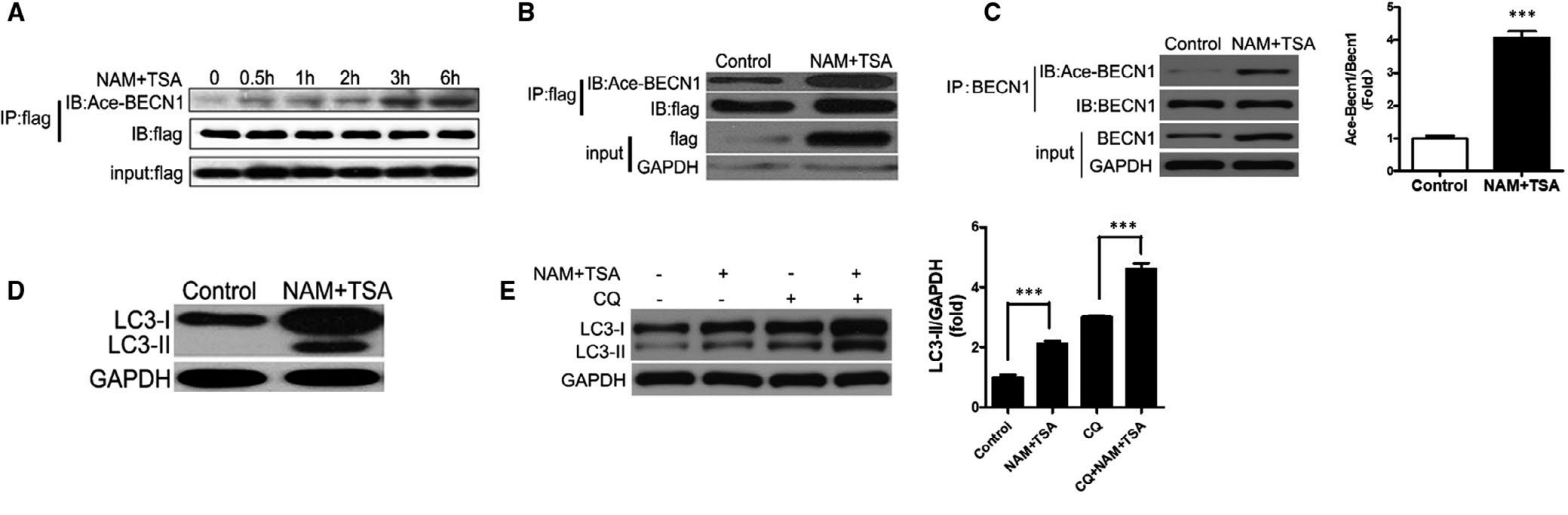
Treatment with NAM and TSA increased the levels of Ace‐BECN1, BECN1 and LC3 both in 293T cells and 3T3‐L1 adipocytes. BECN1 was overexpressed in 293T cells. After treatment with the deacetylase inhibitor nicotinamide (NAM) 5 mM, and the histone deacetylase inhibitor, TSA 10 µM for 0‐6 h, immunoprecipitation (IP) showed elevated levels of acetylated BECN1 (Ace‐BECN1) (A). BECN1 was overexpressed in 293T cells by the lentivirus. After treatment with NAM 5 mM + TSA 10 µM for 12 h, both Ace‐BECN1 and BECN1 levels were elevated (B). BECN1 was overexpressed in 3T3‐L1 mature adipocytes by the lentivirus. After treatment with NAM and TSA for 12 h, both Ace‐BECN1 and BECN1 levels were elevated (C). After 293T cells (D) or 3T3‐L1 mature adipocytes (E) were treated with NAM 5 mM + TSA 10 µM, CQ 60 µM or NAM 5 mM + TSA 10 µM + CQ 60 µM for 12 h, Western blot showed that LC3‐II expression was increased. Data are shown as the means ±standard deviations of three independent experiments performed in triplicate. ^***^ *P* < .001

**FIGURE 3:**
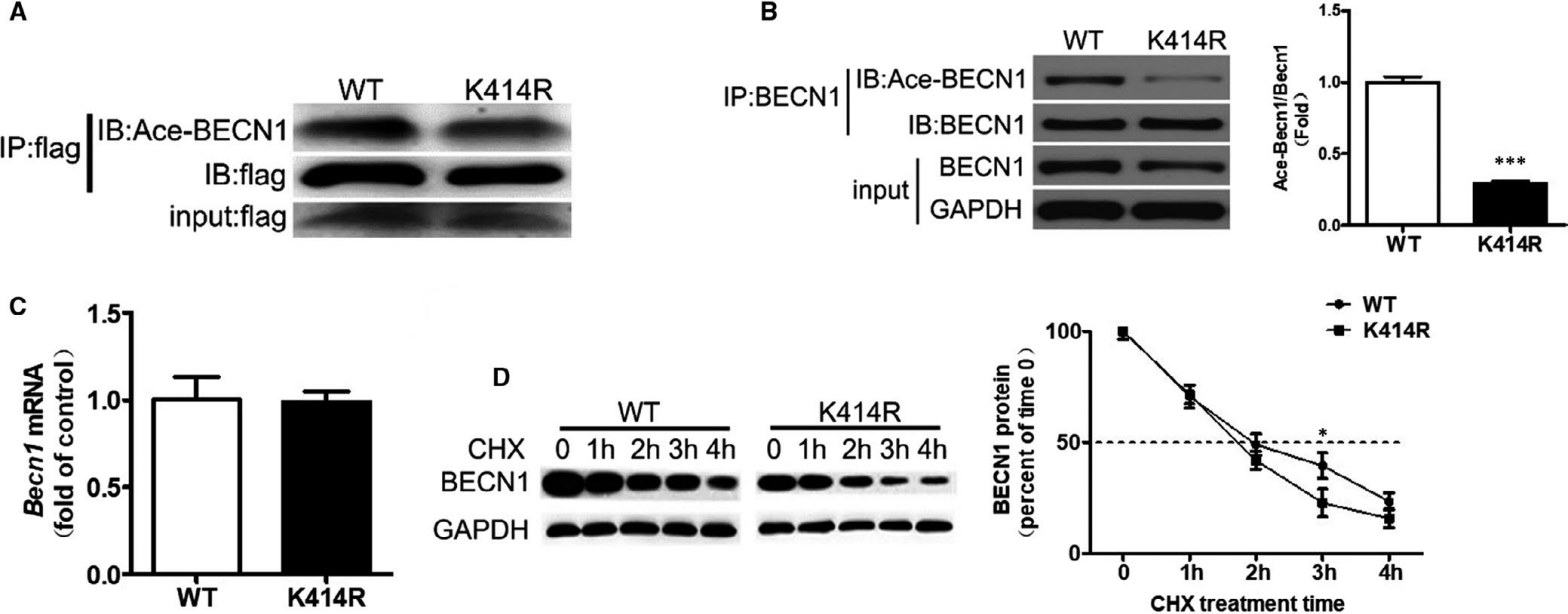
Identification of a new BECN1 acetylation locus. A lentivirus for BECN1 acetylation locus K414R mutation was constructed and used to infect 293T cells (A) or 3T3‐L1 mature adipocytes (B). Immunoprecipitation (IP) showed that the levels of Ace‐BECN1 and BECN1 protein were decreased. The mRNA levels of BECN1 were unchanged after K414R lentivirus infection in 293T cells (C). K414R lentivirus was used to infect 293T cells. The cells were treated with cycloheximide (CHX) 100 µg/mL for 4 h to inhibit protein synthesis. The degradation rate of BECN1 protein was increased, suggesting that BECN1 acetylation altered its protein content by affecting the stability of BECN1 protein (D). Data are shown as the means ±standard deviations of three independent experiments performed in triplicate. ^*^ *P* < .05

BECN1 was overexpressed in 293T cells by the lentivirus. After treatment with the deacetylase inhibitor NAM and the histone deacetylase inhibitor TSA, IP showed that the levels of acetylated BECN1 increased gradually with time (Figure 2A). Following treatment with NAM and TSA for 12 hours, Ace‐BECN1 and BECN1 levels were elevated in both 293T cells (Figure 2B) and 3T3‐L1 mature adipocytes (Figure 2C). Increased LC3‐II expression was found in 293T cells (Figure 2D) and 3T3‐L1 mature adipocytes (Figure 2E) treated with NAM and TSA with/without CQ, as shown by Western blot (Figure 2D). These results suggested that BECN1 acetylation was present in adipocytes, which may affect the expression of BECN1, and thereby further promoted autophagy and autophagy flux.

### 3.3 The K414R mutation affects BECN1 expression

By searching ASEB (http://bioinfo.bjmu.edu.cn/huac/search_p/), K414 was predicted to be the acetylation site of BECN1. We then constructed the BECN1 K414R overexpression lentivirus. The 293T cells were transfected with a lentivirus for BECN1 acetylation locus K414R mutation. IP showed that the levels of Ace‐BECN1 and BECN1 were decreased by the mutation (Figure 3A). After transfection of the mutated BECN1 in 3T3‐L1 mature adipocytes, Ace‐BECN1 and BECN1 protein levels also decreased (Figure 3B). In order to study how the K414R mutation affected BECN1 expression, we examined the mRNA levels of *Becn1*, which were unchanged after K414R lentivirus infection in 293T cells (Figure 3C). We further verified that the K414R mutation accelerated the degradation rate of BECN1 protein in 293T cells when co‐treated with cycloheximide (CHX) to inhibit protein synthesis (Figure 3D). These results suggested that BECN1 acetylation affected its protein content by affecting the stability of the BECN1 protein.

### 3.4 The K414R mutation affects autophagy

**FIGURE 4:**
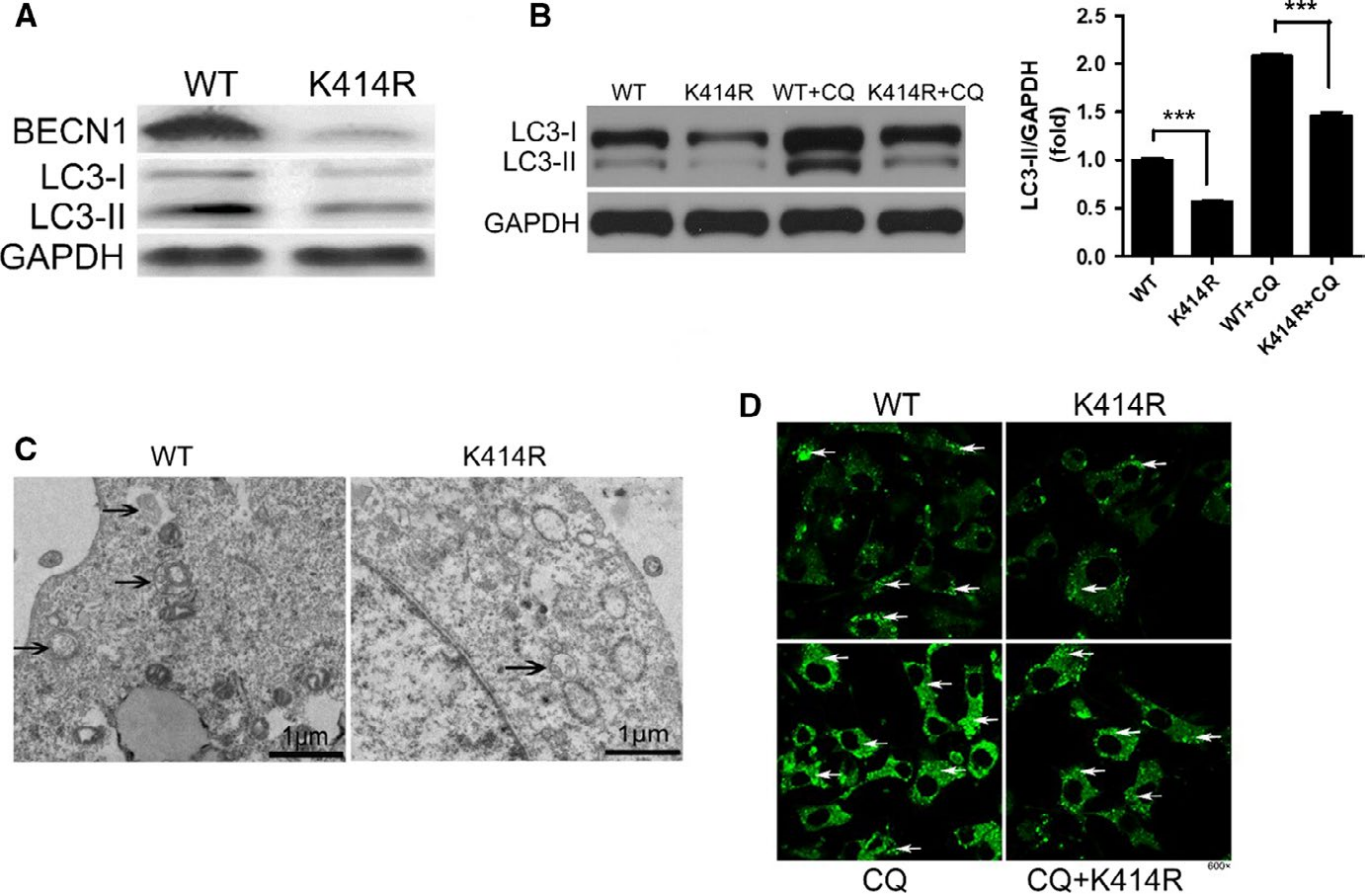
K414R mutation decreases autophagy. K414R lentivirus was used to infect 293T cells (A) or 3T3‐L1 mature adipocytes (B) with/without CQ 60 µM. Western blot showed that the expression of LC3‐II was decreased after K414R treatment. K414R lentivirus was used to infect 3T3‐L1 mature adipocytes. Transmission electron microscopy revealed a significant decrease in autophagosome‐related structures (C). K414R lentivirus was used to infect 3T3‐L1 mature adipocytes. Following treatment with CQ 60 µM for 12 h, the punctiform aggregation of LC3 in the locus mutation group was reduced as shown by the immunofluorescence assay under a confocal microscope (D). Data are shown as the means ±standard deviations of three independent experiments performed in triplicate. ^***^ *P* < .001

The K414R overexpression lentivirus was used to infect 293T cells and 3T3‐L1 mature adipocytes. Western blot showed that the expression of LC3‐II was decreased by the K414R mutation with/without CQ treatment (Figure 4A‐B). Autophagosome‐related structures including sequestering phagophores, typical double‐membrane autophagosomes and autolysosomes were observed by transmission electron microscopy following infection of 3T3‐L1 mature adipocytes with Becn1 overexpression lentivirus (Figure 4C). However, the number of autophagosome‐related structures was decreased by the K414R mutation (Figure 4C). After treatment with CQ for 12 hours, the punctiform aggregation of LC3 in the K414R mutation group was reduced as shown by the immunofluorescence assay (Figure 4D), which suggested that K414R mutation inhibited autophagy flux. Taken together, these results suggested that the K414R mutation inhibited autophagy and autophagy flux in mature adipocytes.

### 3.5 The K414R mutation inhibits adipocyte differentiation

**FIGURE 5:**
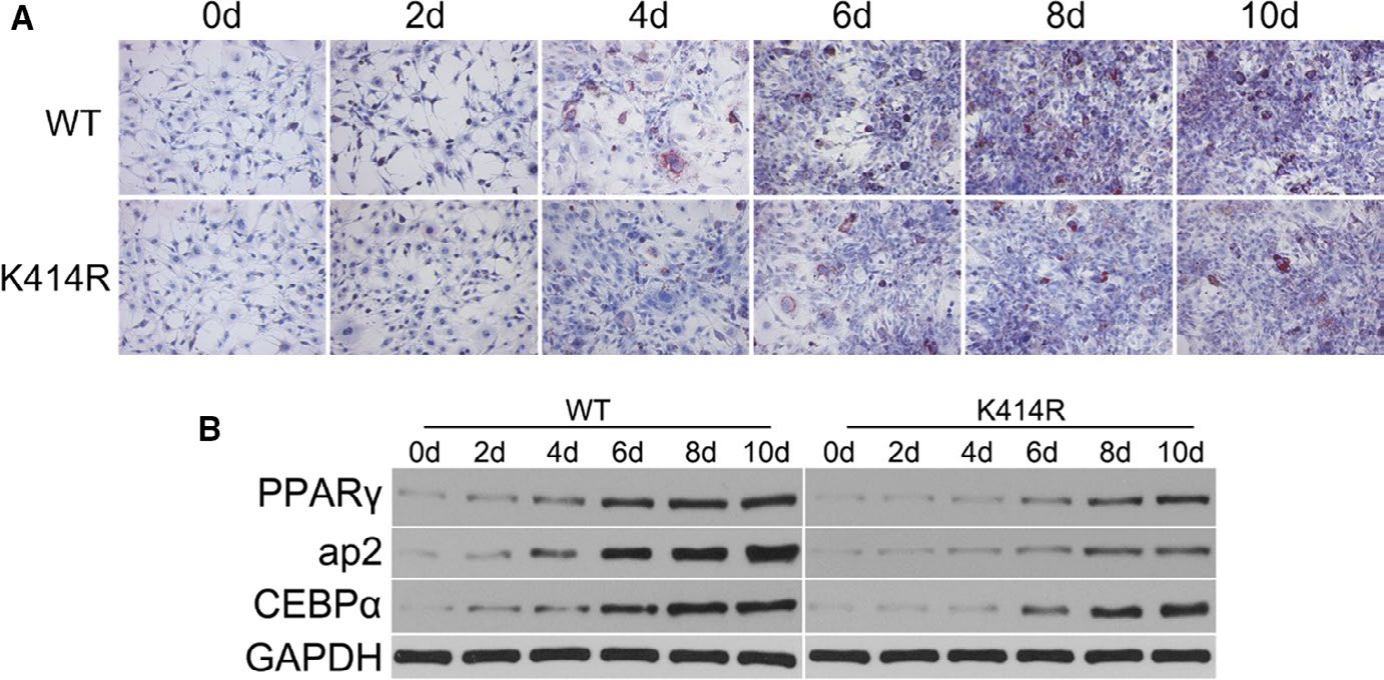
K414R mutation inhibited adipocyte differentiation. K414R lentivirus was used to infect 3T3‐L1 pre‐adipocytes. After induction for up to 10 days, the oil red O staining in the mutant group was reduced, suggesting inhibition of differentiation (A). K414R lentivirus was used to infect 3T3‐L1 pre‐adipocytes. After induction for up to 10 days, the expression levels of the adipocyte differentiation‐related proteins PPARγ, ap2 and CEBPα were decreased compared with those in the control group (B)

The K414R lentivirus was used to infect 3T3‐L1 pre‐adipocytes. After induction for up to 10 days, oil red O staining of the mutant group was reduced, suggesting inhibition of differentiation (Figure 5A). The levels of the adipocyte differentiation‐related proteins PPARγ, ap2 and CEBPα were decreased compared with those in the WT group (Figure 5B).

### 3.6 The K414R mutation inhibits lipolysis

**FIGURE 6:**
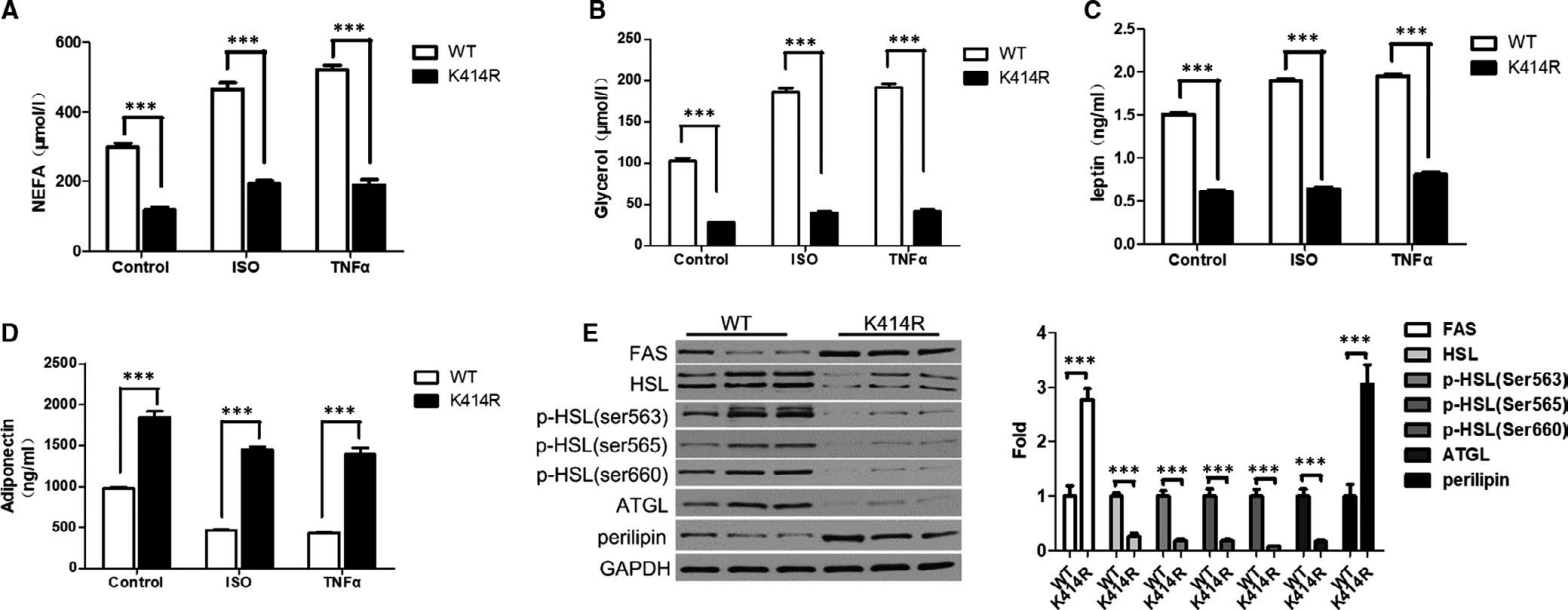
K414R mutation inhibits lipolysis. K414R lentivirus was used to infect 3T3‐L1 mature adipocytes. Following treatment with ISO 1 µM for 30 min and TNF‐α 50 ng/mL for 3 h, a decrease in the content of non‐esterified fatty acids (NEFA) and glycerol in the culture medium of the K414R group was observed (A‐B). K414R lentivirus was used to infect 3T3‐L1 mature adipocytes. Following treatment with ISO 1 µM for 30 min and TNF‐α 50 ng/mL for 3 h, ELISA showed a decrease in the content of leptin (C) and an increase in adiponectin content (D) in the culture medium of the K414R group. K414R lentivirus was used to infect 3T3‐L1 mature adipocytes. Western blot showed that the expression of fatty acid synthase (FAS) was increased, and the expression of the lipolytic enzymes HSL, p‐HSL and ATG was decreased, and the expression of perilipin was increased (E). Data are shown as the means ±standard deviations of three independent experiments performed in triplicate. ^***^ *P* < .001

The K414R lentivirus was used to infect 3T3‐L1 mature adipocytes. After treatment with ISO for 30 minutes and TNF‐α for 3 hours, a decrease in the content of non‐esterified fatty acids (NEFA) and glycerol in the culture medium of the K414R group was observed (Figure 6A‐B). ELISA showed a decrease in the content of leptin in the culture medium of the K414R group (Figure 6C), while there was an increase in adiponectin content in the culture medium of the K414R group (Figure 6D). Western blotting showed that the expression of fatty acid synthase (FAS) was increased, and the levels of the lipolytic enzymes HSL, p‐HSL and ATG were decreased, and the expression of perilipin, a crucial lipid droplet protein, was increased (Figure 6E). Taken together, these data strongly suggested that the K414R mutation inhibited lipolysis in adipocytes.

## 4 DISCUSSION

BECN1 is involved in adipocyte differentiation, lipolysis and insulin resistance. There are no studies available on BECN1 acetylation in adipocytes. Therefore, this study aimed to investigate the impact of mutations at the acetylation sites of BECN1 on adipocyte differentiation and lipolysis. The results strongly suggested that the acetylation of BECN1 was observed in mature adipocytes. The novel, unreported acetylation locus K414 was identified. Acetylation of BECN1 may be involved in the differentiation and lipolysis of adipocytes.

Autophagy is involved in lipid metabolism in hepatocytes, where it modifies lipid droplets.^2^ Autophagy is also necessary for the production of large lipid droplets, which is a hallmark of white adipocytes, while the inhibition of autophagy leads to a phenotype of brown adipocytes, leading to increased lipid oxidation and insulin sensitivity.^3^, ^4^ Autophagy is up‐regulated in the adipose tissue of obese patients and correlates with visceral fat distribution and adipocyte lipid content.^14^ However, Soussi et al showed attenuated autophagic activity in human obesity. These authors suggested that autophagy flux should be examined to reflect the true level of autophagy. LC3 is necessary for the formation of autophagosomes and is commonly used as a marker of autophagy.^15^ Evaluation of the expression of LC3 alone does not reflect activation of autophagic clearance. Thus, in this study, the lysosomal inhibitor CQ was used to determine the autophagy flux levels.

Our previous study showed that BECN1 can regulate autophagy in mature adipocytes and shows increased expression in obese mouse models.^8^ The present study showed that the expression of BECN1 and LC3 increased during differentiation from pre‐adipocytes to adipocytes, supporting the role of BECN1 and autophagy in mature adipocytes. In addition, higher levels of BECN1 and LC3 were found in the adipose tissues of obese mice compared with non‐obese mice.

Acetylation is involved in the regulation of autophagy.^16^, ^17^ There is currently one study on BECN1 acetylation in tumour cells^11^ and one in Alzheimer's disease.^12^ The present study is the first to demonstrate that Ace‐BECN1 is involved in the autophagy of adipocytes. The protein levels of Ace‐BECN1 changed with adipocyte differentiation and lipolysis. We identified K414 as an acetylation site of BECN1, which affects the stability of the BECN1 protein. We found knockdown the endogenous BECN1 inhibited pre‐adipocytes differentiation, which can be rescued by overexpression of BECN1 WT. However, overexpression of BECN1 K414R partly rescued it (Figure S1), which means other acetylation sites of BECN1 may exist and also affect pre‐adipocytes differentiation. In addition to K416, K324 and K206 were predicted to be acetylation sites of BECN1 (*Homo sapiens*). However, we found that K324 and K206 did not affect BECN1 acetylation (data not shown). Other acetylation sites of BECN1 may exist and affect lipid metabolism. Therefore, in the next study, we will use mass spectrometry to screen all the candidate acetylation sites of BECN1, and confirm their function in adipocytes.

The K414R mutation decreased the levels of BECN1 and LC3 (with/without CQ treatment), suggesting that the acetylation of BECN1 affected autophagy of adipocytes. PPARγ, ap2 and CEBPα, which are markers of adipocyte differentiation,^18^ were decreased after K414R mutation. In addition, oil red O staining of the mutant group was reduced, suggesting inhibition of differentiation. In addition, the K414R mutation significantly inhibited lipolysis in adipocytes. Lipolysis is a biochemical catabolic pathway, in which LD‐associated lipases, such as adipose triglyceride lipase (ATGL) and hormone‐sensitive lipase (HSL), are activated. In addition, the interaction with LD‐associated proteins, such as perilipin, was also verified to regulate lipolysis. Decreased expression of the lipolytic enzymes HSL, p‐HSL and ATGL, and increased expression of perilipin were found after disruption of BECN1 acetylation. Our previous study found that TNFα‐induced autophagy can selectively degrade perilipin 19; therefore, the autophagic clearance of proteins associated with lipolysis may be a new way to regulate lipid metabolism. Adipose tissue is now recognized as an important endocrine organ, secreting a large number of endocrine factors, such as adiponectin and leptin. Adiponectin induces cytotoxic autophagy in breast cancer cells^20^ and stimulates autophagic flux in cultured skeletal muscle cells.^21^ In adipocytes, autophagosome dynamics are moderately enhanced by leptin.^22^ Most studies have shown that adiponectin and leptin regulate autophagy, but few studies have focused on how autophagy affects adiponectin and leptin levels. Slutsky et al found decreased adiponectin and increased leptin secretion in cultured adipocytes stimulated with TNFα+IL‐1β, which was partially reversed by small interfering RNA‐mediated knockdown of ATG7.^23^ In the present study, the K414R mutation decreased the secretion of leptin and increased the secretion of adiponectin by adipocytes. These findings indicated that autophagy may regulate adiponectin and leptin secretion. It is considered that ER stress can promote autophagy‐dependent adiponectin degradation in 3T3‐L1 adipocytes.^24^ Thus, it is possible that K414R mutation inhibits autophagy, thereby decreasing adiponectin expression. More studies are needed to determine how BECN1 acetylation affects adipocytokine expression and secretion.

These results suggested that modulating the acetylation of BECN1 may be used to alter the phenotype of adipocytes and alleviate their role in the development of obesity, T2DM and its complications. Indeed, deacetylase inhibitors induce autophagy,^25^ while acetylation by acetyltransferase p300 inhibits autophagy.^16^ The deacetylase and acetyltransferase of BECN1 should be examined more closely in future studies.

## CONFLICT OF INTEREST

We do not have any conflicts of interest to declare.

## AUTHOR CONTRIBUTION

Each of the authors acknowledge that he or she participated sufficiently in the work to take public responsibility for its content.

Chengqian Li: Project administration and investigation. Jun Xu: Funding acquisition; writing the original draft; investigation; reviewing and editing the manuscript. Qing Yu: Methodology; data curation; investigation; visualization; reviewing and editing the manuscript. Ping Wang: Software; investigation; data curation; validation. Bingzi Dong: Funding acquisition and investigation. Liyan Shen: Project administration and resources. Qing Wang: Investigation and visualization. Shufa Li: Conceptualization and methodology. Ying Yang: Conceptualization. Yujie Deng: Conceptualization; supervision; funding acquisition; investigation.

## ETHICS APPROVAL

The experimental protocol was approved by the Ethics Committee for Animal Experimentation of the Affiliated Hospital of Qingdao University.

## Supporting information

Fig S1
