# Supplementary figures and images for: Mutation of the novel acetylation site at K414R of BECN1 is involved in adipocyte differentiation and lipolysis
Source: J Cell Mol Med. 2021 Jun 4;25(14):6855–63. doi: 10.1111/jcmm.16692 (PMC8278081; doi:10.1111/jcmm.16692)

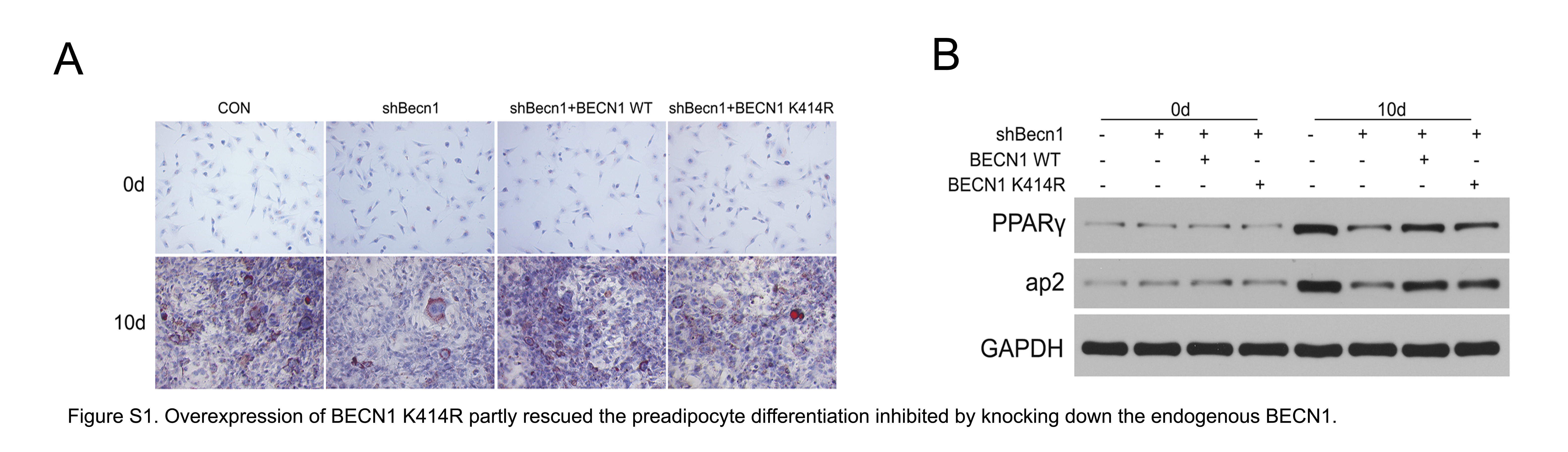

Supplement: Supplementary file 1 — Fig S1 [file JCMM-25-6855-s001.tif]
